# Encoder-Controlled Functional Electrical Stimulator for Bilateral Wrist Activities—Design and Evaluation

**DOI:** 10.3390/bioengineering9100501

**Published:** 2022-09-24

**Authors:** Cassandra D. Solomons, Vivekanandan Shanmugasundaram, Sivakumar Balasubramanian

**Affiliations:** 1 Department of Instrumentation and Control, School of Electrical Engineering, Vellore Institute of Technology, Vellore 632014, Tamil Nadu, India; 2 Department of Bioengineering, Christian Medical College and Hospital, Bagayam, Vellore 632002, Tamil Nadu, India

**Keywords:** bilateral wrist training, functional electrical stimulation, angle controlled stimulation, stroke rehabilitation, brain plasticity (Min.5-Max.8)

## Abstract

Upper limb impairment following stroke is often characterized by limited voluntary control in the affected arm. In addition, significant motor coordination problems occur on the unaffected arm due to avoidance of performing bilateral symmetrical activities. Rehabilitation strategies should, therefore, not only aim at improving voluntary control on the affected arm, but also contribute to synchronizing activity from both upper limbs. The encoder-controlled functional electrical stimulator, described in this paper, implements precise contralateral control of wrist flexion and extension with electrical stimulation. The stimulator is calibrated for each individual to obtain a table of stimulation parameters versus wrist angle. This table is used to set stimulation parameters dynamically, based on the difference in wrist angle between the set and stimulated side, which is continuously monitored. This allows the wrist on the stimulated side to follow flexion and extension patterns on the set side, thereby mirroring wrist movements of the normal side. This device also gives real-time graphical feedback on how the stimulated wrist is performing in comparison to the normal side. A study was performed on 25 normal volunteers to determine how closely wrist movements on the set side were being followed on the stimulated side. Graphical results show that there were minor differences, which were quantified by considering the peak angles of flexion and extension on the set and stimulated side for each participant. The mean difference in peak flexion and extension range of movement was 2.3 degrees and 1.9 degrees, respectively, with a mean time lag of 1 s between the set and the stimulated angle graphs.

## 1. Introduction

Stroke is a major cause of impairment and disability in the world. A large share of the global disease burden stems from the disability caused by non-communicable diseases as compared to death. This has only progressed, from 21% of the burden in the 1990s to 34% by 2019. In adults, between 1990 and 2019, stroke was the third contributor after ischemic heart diseases and diabetes mellitus to increasing health loss measured as increase in disability-adjusted life years (DALYs) by 32% [1].

Rehabilitation of the upper extremity primarily focuses on functional recovery of the paretic arm. Common methods used for the rehabilitation of the paralyzed arm and hand are passive exercises to normalize tone and prevent contractures and, thereafter, active exercises once partial or full voluntary control is achieved. However, research shows that bilateral training should be added to the normal therapy regime since it offers significant benefits [2,3,4,5] Improved results are seen when both limbs are involved in the exercise, rather than exercising the impaired limb alone [6,7,8]. Kelso et al. studied the movement time, velocity and acceleration patterns generated when performing bilateral synchronous movements with a mix of easy and difficult targets. They showed that though reaction time between the two upper limbs might vary based on the complexity of the target, the velocity and acceleration patterns were synchronous, suggesting that there is central coordination of muscle activity rather than independent limb control [9].

Bilateral training devices are of many types, ranging from very simple pulleys or mirrors to large mechanical devices. Stoykov et al. describe a few simple bilateral training devices [10], and Delden et al. have reviewed mechanical and robotic devices that have been used in recent years for bilateral arm training [11]. Brackenridge et al. reviewed 141 mechanical and robotic devices for stroke and concluded that an effective device is any simple device that can promote neuroplasticity and does not have to be unnecessarily complex and expensive [12].

A stroke is a neurological event that compromises the supply of blood in the brain, which causes damage to the neural tracts in the affected area. Therefore, when stroke impacts the regions of the brain that control arm movement, it can cause weakness or paralysis in the contralateral arm. The primary pathology is in the brain, when usually there is nothing wrong with the arm itself. Some disuse muscle atrophy may occur with an element of increased tone in the affected arm. Thus, recovering arm movement after stroke revolves around healing the brain and rebuilding neural pathways [13]. This is possible through neuroplasticity, which is the brain’s natural ability to rewire itself and learn new skills or re-learn old skills. Although severely damaged brain cells may not recover, the brain is surprisingly adaptive. It can recruit the physiologically intact surrounding neural cells to take on lost functions through neuroplasticity. The brain can be encouraged to adapt through repetitive practice of a skill or movement [14]. This reinforces these neural pathways to become stronger.

Evidence suggests that the stroke-affected brain is highly adaptable and, if given the correct motor strategies, can be reshaped in a constructive way [13]. Electrical stimulation is one of the modalities that encourages this type of plasticity in neurons of the stroke-affected brain [15,16,17,18]. Electrical stimulation in arm rehabilitation has been used widely to retrain functional properties of the musculoskeletal units and replace function. When electrical stimulation is applied through the electrodes, the current causes the muscles to contract, resulting in movement. Electrical stimulation that uses the correct stimulation parameters on appropriate muscle synergy during the optimal therapeutic window has proven to impart positive re-learning benefits [15]. The superiority of functional electrical stimulation (FES) over conventional therapy is yet to be proved, but pairing electrical stimulation with conventional therapies is ongoing and has a promising future [19].

Electrical stimulation combined with bilateral arm training has been shown to improve voluntary control in stroke patients, besides reducing neurological deficits [20,21,22,23]. When contralateral control was included with bilateral hand activities, there was a significant improvement in joint range of motion (ROM) [22] and shortening of treatment time in acute stroke patients [24].

Some devices have been designed for bilateral control of stimulation to the stroke-affected arm. The command glove, designed for finger extension with the help of EMG feedback from the contralateral hand, is an example of such a device [22]. It uses an instrumented glove and feedback from sensors to set stimulation parameters. Ka-leung Chan et al. used FES with bilateral training tasks; FES was triggered by the movement of a finger [25]. Similar trials were performed with FES on normal subjects, for bilateral control of the elbow joint [26]. The authors reported that the trial was unsuccessful due to larger loop current interference in signals between the muscles.

Mirror therapy is being used successfully in stroke rehabilitation for recovery of lost function. It is applied by placing the stroke-affected limb within a mirror box so as to completely hide the limb from vision. The reflection of the normal side appears to be the stroke affected limb to the user and any activity performed by the normal side appears to the user as being performed by the stroke-affected side. Studies have shown that mirror therapy has influenced re-learning of activity in the brain [27,28,29], and mirror therapy combined with electrical stimulation is even more beneficial [30,31,32].

This paper describes the design of an encoder-controlled FES that uses electrical stimulation to allow precise control of wrist flexion and extension and coordinate bilateral wrist activity with the help of feedback from encoders. This low-cost device continuously monitors the difference in angle between both wrists and determines the stimulation parameters to be fed into two channels of the stimulator, so as to stimulate the wrist on one side to match the angle of the other. In real-time, it follows the wrist movements of the normal side, so as to act as a mirror refection of it. Here, the mirror is replaced by actual mirroring movement activity with the help of functional electrical stimulation. The device is intended to mimic mirror therapy without a mirror, since the user can actually visualize the stroke-affected wrist moving synchronously with the normal wrist. As a first step to introduce this device into therapeutic rehabilitation, it was decided to test it for efficiency on healthy volunteers.

## 2. Materials

The experimental set-up consisted of an eight-channel functional electrical stimulator, of which two channels were used—one for wrist flexion and the other for extension. Two adjustable and moveable pedestals were used to carry the encoders and related circuitry that were housed in two 3D-printed boxes. The pedestals also served to hold the armrest and the encoder handles. A detailed description of the hardware involved follows.

### 2.1. Electrical Stimulator

The muscle stimulator used in the study is an 8-channel electrical stimulator designed in the Bioengineering Department of Christian Medical College and Hospital, Vellore, India [33]. This is a portable device that has been used for functional electrical stimulation in various departments of the hospital. It could be used standalone, by pre-programming the stimulation parameters for each of the channels, or connected to a computer and instantly setting the parameters via the serial port. Each of the channels is programmed to deliver pulses of the desired frequency, amplitude and pulse width by sending 1 byte of data that carries this information. Of this 1 byte, 2 bits are used for setting frequency (5 Hz, 10 Hz, 20 Hz and 40 Hz four frequencies in all), and 3 bits each for amplitude (15 mA to 40 mA, eight in all) and pulse-width (80 µs to 240 µs, eight in all) of the stimulation pulses. In addition, 1 byte of data is used for turning the stimulator on and 1 byte for turning the stimulator off. For this study, the first two channels, namely channel 1 and channel 2, were used. The frequency of stimulation pulses was fixed at 40 Hz, since good tetanic contraction was observed at this frequency, and the other parameters were instantly set based on feedback.

### 2.2. Encoder Circuit

Two orange three-phase incremental optical rotary encoders, whose output was compatible with Arduino using pull-up resistors, were used to detect the change in wrist angle on both sides. One of the encoders recorded wrist angles on the set side and the other on the stimulated side. The Arduino Due was chosen since it has four serial ports, two of which were used—one to write to the serial port of the computer for further processing in Matlab, and the other to write to the Rx pin of the stimulator for changing stimulation parameters. The stimulator was optically isolated from the rest of the circuitry, which included the Due board, encoders and the computer. A PCB, carrying the isolation circuit and the encoder with pull up resistors and other related circuitry, was made to fit over the Duo board like a shield.

### 2.3. Pedestal-Mounted Encoder Circuit

**Figure 1:**
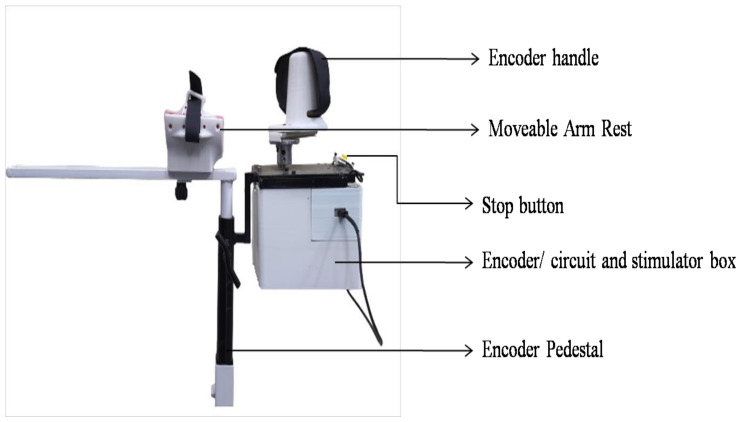
Pedestal-mounted encoder set-up. The figure shows the arm rest and encoder handle, below which is a box holding the base of the encoder, related circuitry and stimulator enclosure

**Figure 2:**
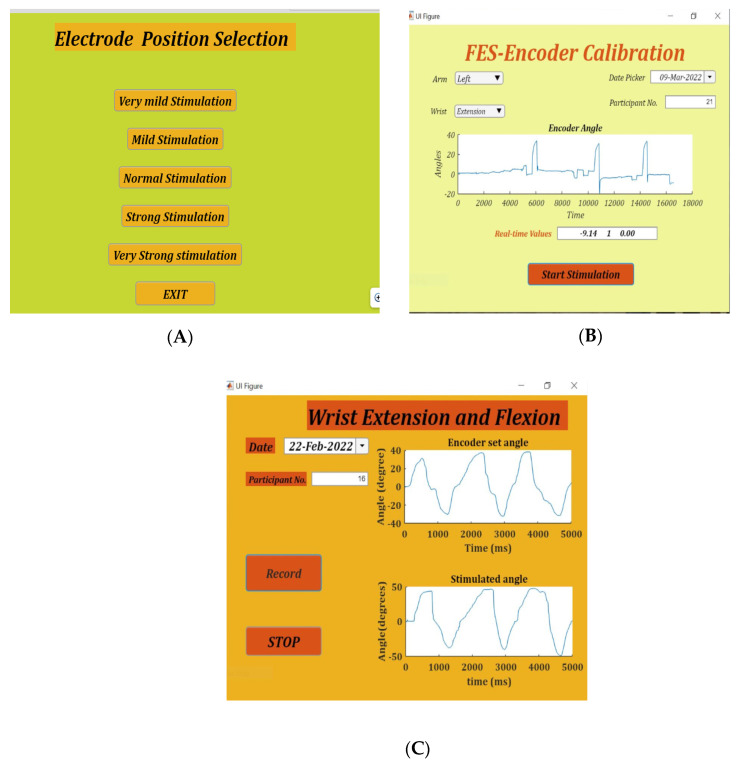
The three apps, namely. (**A**): Position Selection—used to determine the correct position of flexion/extension electrodes. (**B**): FES-Encoder Calibration—for obtaining the flexion/extension calibration tables, and (**C**): Set-stimulate Record angles app—for final recording of wrist set and stimulate angles with real-time feedback.

Two custom-made pedestals, adapted from the PLUTO height-adjustable trolley used in [34], were used to hold the encoders and their related circuitry and served as armrests for the user. Encoder boxes, designed in Solidworks 2020 and 3D-printed, housed the two encoders, related circuitry and stimulator, as shown in Figure 1. The output shafts of the encoders needed to be lengthened, and this was done by screwing the shafts onto a longer cylindrical hollow tube, such that the movement of the shaft was transferred to this tube. The tube was then brought out from the underside of the top laser-cut plate with the help of a bearing, thereby representing the shaft of the encoders. Linear guides were screwed onto universal mounts, which were in turn connected to the encoder shafts. The 3D-printed encoder handles, adapted from [34], could be slipped into the linear guides. The encoder housing assembly was suspended within the circuitry housing box, with a metal plate and screws. Connections to the computer and stimulator were made via connectors provided on the box. The whole experimental set-up is shown in Figure 2.

### 2.4. Device Firmware

The Arduino Due, a microcontroller based on the Atmel SAM3 × 8E ARM Cortex-M3 CPU, was used to acquire data from the two encoders, transfer the modified stimulation parameters to the stimulator, and enable serial communication with the stimulation software and bidirectional data communication with the custom-made Encoder/Stimulator software via the USB serial port. The encoders gave 4096 transitions per rotation between their outputs. Hence, the firmware calculated the angular position of the shaft using the following formula:

ϴEn = EnV * (360/4096)

ϴEn = EnV * 0.087

where

ϴEn = Angular position of the encoder

EnV = Value read from the Encoder

In the calibration mode, the firmware sends data to alternately change stimulation parameters and stop stimulation at a fixed interval of time (every 6 s). Simultaneously, the angle recorded on the calibration encoder is sent to the device software.

When both the encoders are in use in the set-stimulate mode, the firmware calculates the difference between the encoder readings and converts it to an angle using the following formula:

ϴ_En_ − ϴ_EnSTIM_ = (EnV − EnV_STIM_) * 0.087

where

ϴ_En_ = Angular position of the encoder that sets the angle on the normal side

EnV = Value read from the encoder on the normal side

ϴ_EnSTIM_ = Angular position of the encoder on the stimulation side

EnV_STIM_ = Value read from the encoder on the stimulation side

This difference in angle is sent to the device software, based on which it returns a byte of data for stimulation parameter change to the stimulator via the firmware.

### 2.5. FES-Encoder Software

MATLAB App Designer (Mathworks Inc. Version R2021b) was used to create the apps for user interaction. The apps present a graphical user interface (GUI) for the user and, via the firmware, receive information on the difference in angle of the encoders and simultaneously send stimulation parameter change information to the stimulator. The angles recorded and the change in stimulation parameters are stored and presented as a graph for further analysis. The main FES-Encoder app provides the interface to choose from three sub-apps, namely.

### 2.6. Placement Selection App

FES-Encoder Calibration app and Stimulate and Record Angles app. Placement selection app:

Finding the appropriate position of the electrodes is achieved by using the placement selection app. This app gives five combinations of stimulation amplitudes and pulse width, namely very mild stimulation (15 mA and 80 µs), mild stimulation (15 mA and 120 ms), normal stimulation (15 mA and 170 ms), strong stimulation (18 mA and 120 ms) and very strong stimulation (18 mA and 200 ms). On selection of any of these modes, stimulation pulses are delivered for 8 s. Figure 2A shows the placement selection app.

### 2.7. FES-Encoder Calibration App

The FES-Encoder calibration app requires the user to enter whether the left or right hand is being calibrated and whether the flexors or extensors are being stimulated. Pressing the “Record” button on non-selection of these details generates an alert signal asking for the details to be entered. The angle values recorded in the table accordingly are either positive or negative, depending on the position of the encoder handle with respect to the baseline. On pressing the record button, stimulation starts with the lowest amplitude and pulse width and lasts for 4 s, after which there is a 4 s break before the next burst of stimulation with increased pulse width. The app provides a real-time graphical display of the encoder angle when stimulation starts.

**Figure 3:**
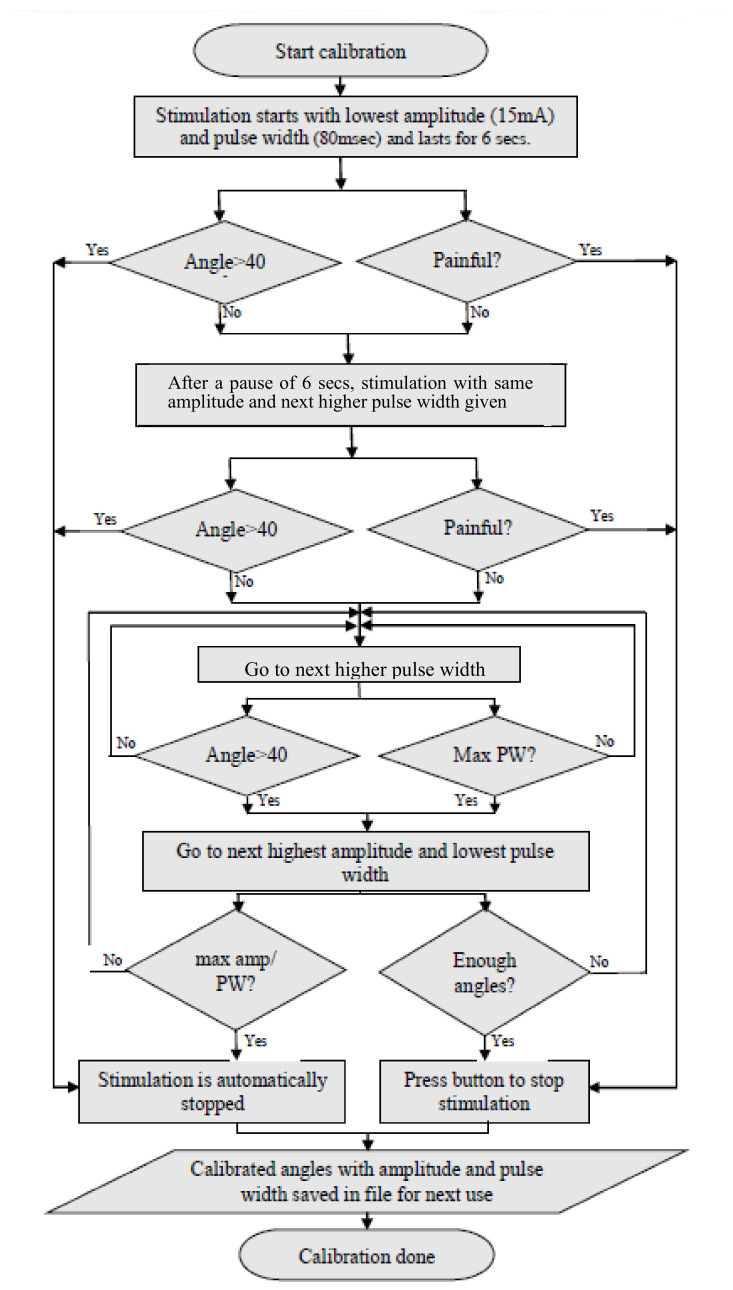
Algorithm followed by the calibration app. Calibration is performed to create the table for use during stimulation mirroring.

On recording an angle of over 40° on the encoder, the stimulator is programmed to skip the remaining pulse widths for that amplitude, go to the next highest amplitude and start over from the first pulse width. This is done to obtain the maximum number of angles with all possible permutations and combinations of stimulation amplitudes and pulse widths. Stimulation is stopped automatically when the lowest pulse width at a given amplitude records an angle over 40°. During the calibration cycle, if painful, the participant can manually stop stimulation with the help of the push button provided. When stimulation is stopped, either manually or by the app, a consolidated graph showing the encoder angles, amplitude and pulse width of stimulation pulses is displayed. Calibration stores the recorded angles in ascending order with the corresponding amplitude and pulse width in the form of a look-up table in two separate files—one for flexion and the other for extension. These angles are used in the set/stimulate and record angles app. Figure 2B shows the FES-encoder calibration app soon after calibration. The calibration cycle is further explained in Figure 3.

### 2.8. Set/Stimulate and Record Angles App

The set/stimulate and record angles app uses the calibration tables that have been saved in a file during stimulation calibration. All angles below the baseline and above 60° are eliminated so as to maintain comfort of stimulation. If there are any two consecutive angles that are within 3° difference of each other, the one with the higher pulse width is removed from the table. Therefore, the look-up table is edited to remove repetitions and avoid confusion when consecutive angles are too close to each other.

On pressing the record button, the current angles on both the encoders are set to 0°. When the handle of the set side encoder is moved, the flexion/extension immediately starts. If the angle of the encoder on the set side is E_SET_ and on the stimulate side is E_STIM_:

E_SET_ = E_STIM_

**Figure 4:**
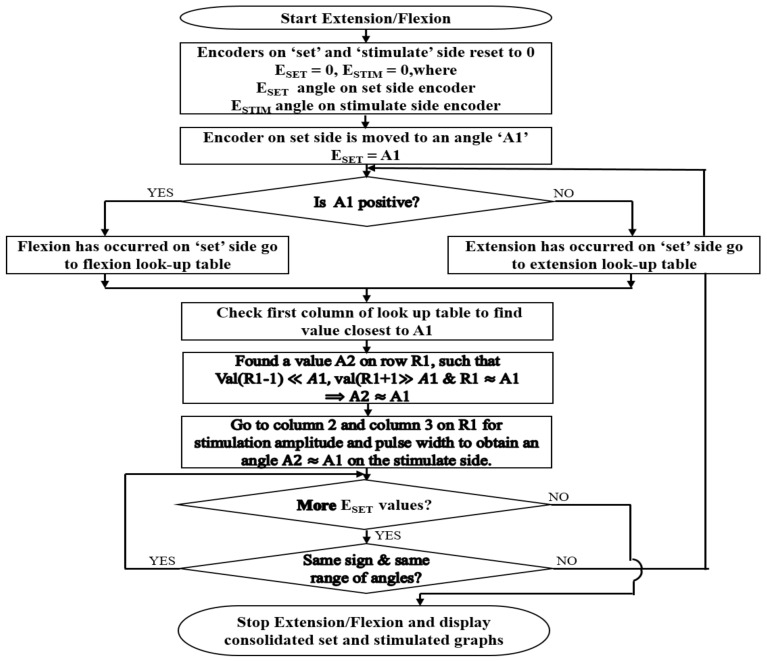
Algorithm followed by the set/stimulate record app. The Flowchart describes how mirroring is performed with the help of the flexion and extension calibration table values.

The magnitude of ESET determines at what angle the stimulated wrist is to be stimulated, and the sign determines the direction of stimulation i.e., whether flexion or extension. The look-up table, obtained during calibration, is used to check the stimulation parameters required to obtain an angle of ESTIM. These parameters are written to the serial port and, via the firmware, reach the stimulator, where the parameters are changed accordingly. Continuous monitoring of the set angles, stimulation angles and changing of stimulation parameters occur for the number of samples specified, after which the stimulator is turned off. A real-time graph on the app page shows the angles of both of the encoders in real time. A consolidated graph of the angles of both encoders along with the change in stimulation parameters is displayed soon after. Figure 4 below shows the operation of the set/stimulate record app.

## 3. Method

### 3.1. Participants

**Figure 5:**
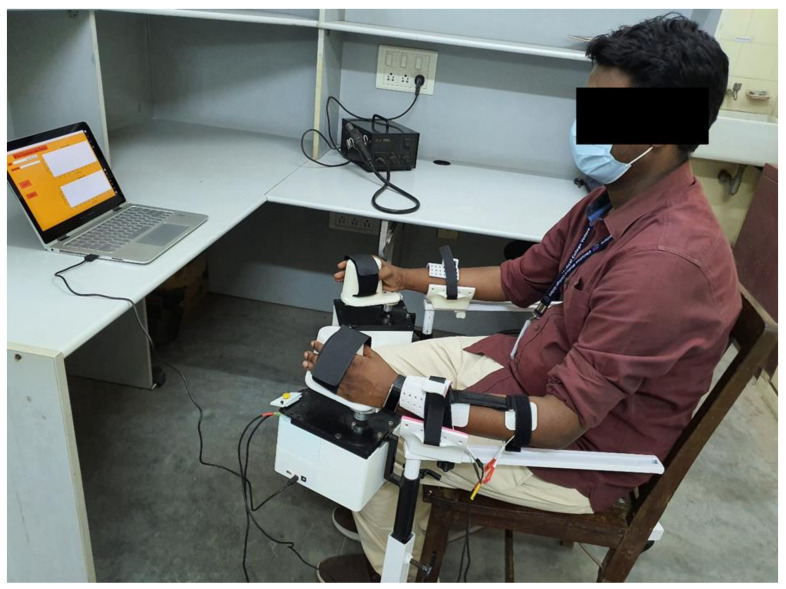
Experimental setup. The figure shows a volunteer using the encoder-controlled FES. The angles are set on the righthand side and the left wrist is stimulated to the same degree of flexion/extension as the set side.

**Figure 6:**
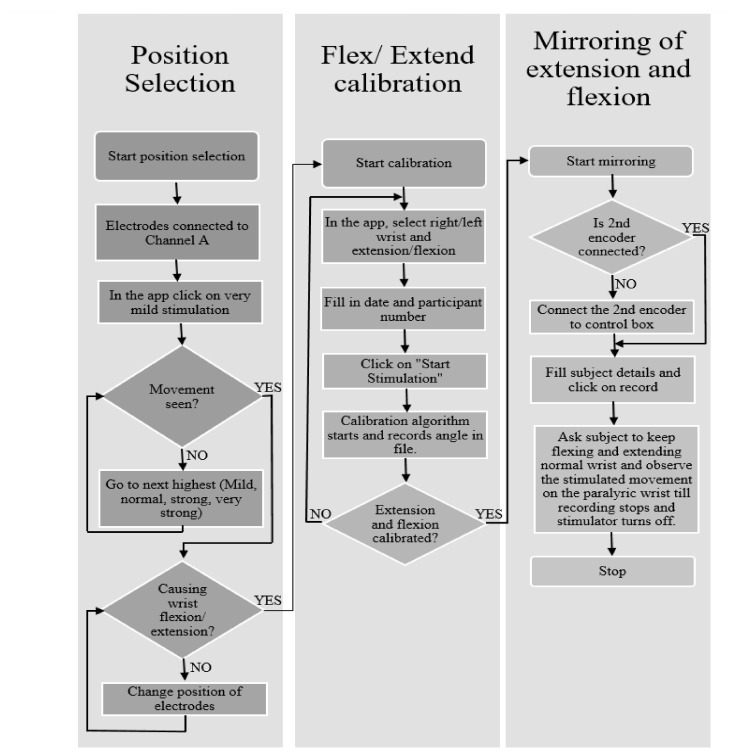
Algorithm to be followed for using the three apps. The Flowchart gives the details on how data were collected from each participant.

The study included 25 (12 female and 13 male) normal volunteers, aged between 18 and 50 years, from among the staff and students of Christian Medical College (CMC), Vellore and Vellore Institute of Technology (VIT), Vellore. The institutional review board of CMC Vellore (IRB Min. No. 14211 [INTERVEN] dated 25 August 2021) and VIT Vellore (Ref. No. VIT/IECH/007(a)/3 March 2020 dated 5 March 2020) approved this study. Informed consent was obtained from all 25 participants. In all participants, the left wrist was stimulated and calibrated. The participants were advised to keep their left arms relaxed throughout stimulation. The whole trial on each subject lasted for about 30–45 min. The subject was asked to sit comfortably on the chair provided and was given a run-through of the three apps. Electrodes were placed on the left extensors—cathode near the motor point and the anode distal to the motor point. The whole experimental setup is shown in Figure 5. The Flowchart showing the algorithm followed throughout the experiment is shown in Figure 6.

### 3.2. Placement Selection

The placement selection app was used to check whether the electrodes were placed correctly. Positioning of the stimulating electrode close to the motor point of the muscle is essential to ensure efficient contraction of the targeted muscle while maximizing comfort during stimulation [35,36,37,38]. Due to variations in the size and shape of the forearm, it was challenging to find the accurate placement of electrodes on the motor point; this involved some time for multiple trials to obtain the best position. The electrodes were placed on the wrist flexors/extensors of the subject’s left arm (in the case of stroke patients, this should be the paralyzed arm) and each of the modes, starting from the very mild stimulation mode, were tried. The mode that produced visible movement of the wrist was used for checking placement of electrodes. Electrodes are moved around till isolated flexion/extension of the wrist is achieved.

### 3.3. FES-Encoder Calibration

The stimulation characteristics of individuals varied based on thickness of the skin, amount of adipose tissue, muscle, placement of electrodes and many other factors [39,40,41]. Once the participant is comfortably seated, the arm is comfortably placed within the armrest of the encoder pedestal, in the neutral position, after finding the correct placement of electrodes. The participant is asked to either grasp the encoder handle or place the palm of the hand on one side of the handle, and it is strapped into position.

On starting the app, the immediate position on the encoder is reset to 0°, and stimulation starts at the smallest amplitude and pulse width. The maximum angle on the encoder for each change in amplitude and/or pulse width is recorded. These angles are stored in a file in the form of a look-up table along with the corresponding amplitude and pulse width in ascending order for future use. An example of the look-up table is shown in Table 1. The software sets the maximum angle of flexion/extension as 40°; however, angles beyond 60° are also seen in the table since a small change in either amplitude or pulse width can sometimes result in a large change in movement.

### 3.4. Set/Stimulate and Record Angles

When the flexion and extension calibration tables are ready, the setup is ready for mirroring the movements of the right wrist onto the left wrist. The normal side encoder pedestal is rolled onto the right side, and the right arm is placed on its armrest. The participant is asked to grasp the encoder handle on the right side with his/her right hand, and it is strapped into position. The normal side encoder is plugged into the place provided on the placement/calibration encoder box on the left side, as shown in Figure 5. Both the encoder pedestals are adjusted according to the comfort of the participant.

The participant is asked to keep the left arm relaxed while moving the handle of the normal side encoder. As the participant moves the wrist on the right side, the stimulation starts on the left side, with the hand moving at the wrist joint thereby mirroring the activity on the right side.

## 4. Results

### 4.1. Placement Selection

Out of the five modes on the placement selection app, the mild stimulation mode was the most commonly used. In very few subjects with less obvious movements, the other modes, normal and strong stimulation, were used. It was easy to find placement of flexion/extension electrodes in 15 participants, but in 3 participants (Nos. 3, 17 and 20), it took about 15 min to decide on the placement of electrodes. In the remaining seven participants (Nos. 1, 4, 12, 13, 14, 21 and 23), finding the correct placement took about 5 to 10 min.

### 4.2. FES-Encoder Calibration

**Figure 7:**
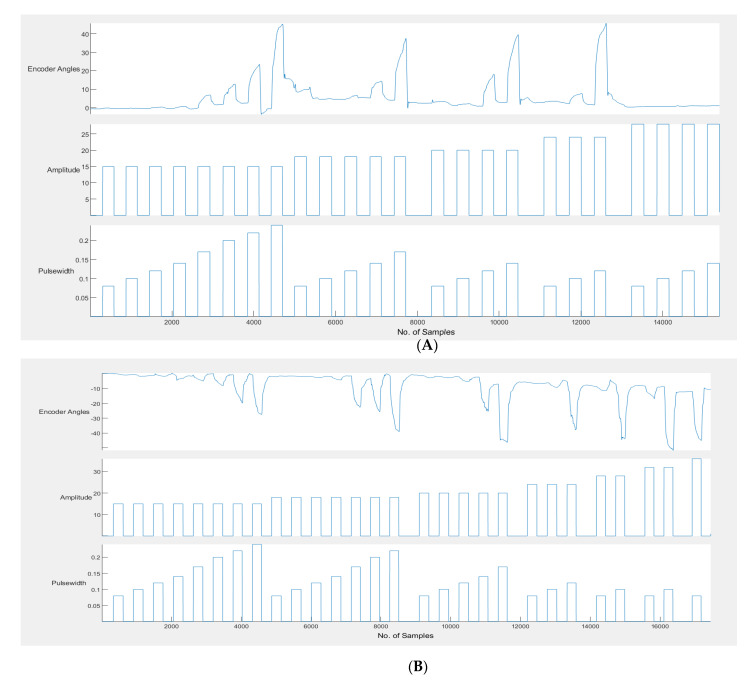
(**A**) Example calibration graph for left extension. (**B**) Example calibration graph for left flexion. The encoder angles obtained for each increase in amplitude and pulse width.

Most of the participants received a wide range of angles within the first three amplitudes (15 mA, 18 mA and 20 mA); an example consolidated graph is shown in Figure 7. It can be seen from Table 1 that the first two encoder angle values were very small negative values instead of 0. This being a left extension required that the angle values be positive and, therefore, these values on the extension table were undesirable. The first two parameters of stimulation did not cause any visible movement, as well. Therefore, they were eliminated from the table. The angle values were rounded off to the nearest 0 so that the first value would be angle 2 with the stimulation parameters of 15 mA and 120 µs. The second, third and fourth values were rounded to 3, but all of them will be eliminated since there is less than a 2 degree difference between the first and these values. In the table, the eighth and ninth value result in an angle of 5 degrees, so the one with the lower pulse width is chosen (i.e., 5, 24 mA, 80 µs), and so on. Therefore, the table is now reduced to one with eight values.

### 4.3. Set/Stimulate and Record

**Figure 8:**
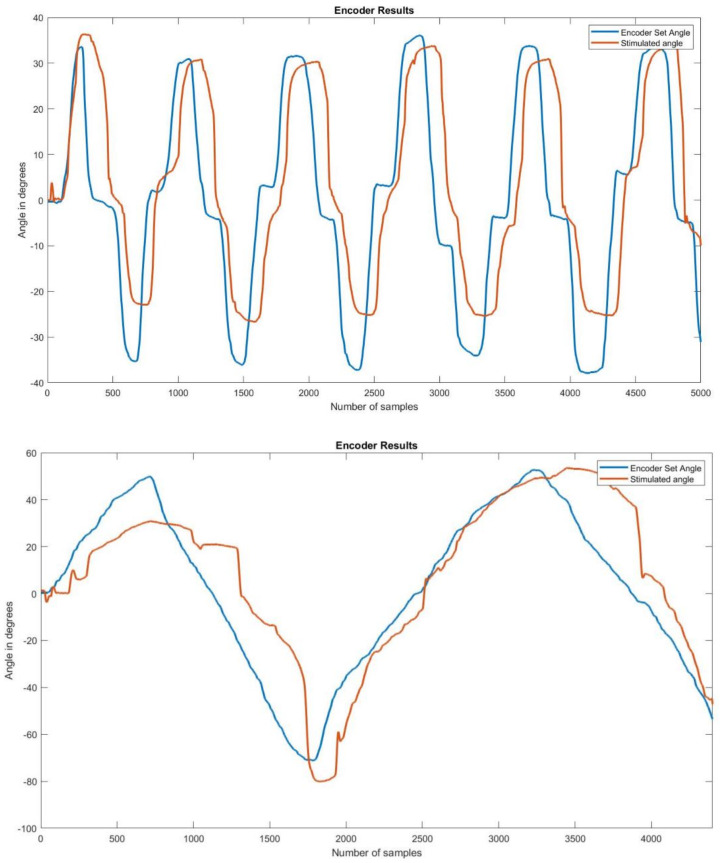
Encoder set and stimulated angle graphs on two participants. Each of the graphs above shows the stimulated angle overlapping the set angle, giving an idea as to how closely they followed each other.

Participants underwent at least two trials of the 50 s stimulate and record procedure. The angle recordings on the left and right encoders showed that the stimulated angle on the left side was following the set angle on the right side. Figure 8 shows two examples of set and stimulated angles in participants.

To check the difference between the set and stimulated angle graphs, the peak extension and peak flexion angles on the set and stimulated sides were compared for the data obtained from all participants. There was a time lag between the set and stimulated angles. The mean time lag was calculated to be 1.02 s.

The graphs above show the difference in peak angles for set and stimulated flexion and extension for each of the participants, and Table 2 gives the mean and standard deviation of the peak angles of flexion and extension for the set and stimulated wrists. The mean differences between the set and stimulated wrists were 2.4 and 1.9 for flexion and extension, respectively. The mean time lag between the set and stimulated angles was 1.02 s. The actual values for peak flexion/extension set and stimulated values are provided as a table in supplementary data.

**Figure 9:**
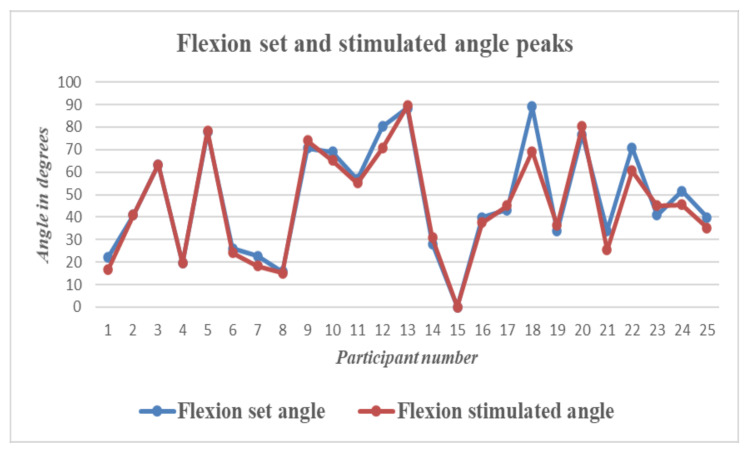
Wrist Flexion—Graph showing differences between set and stimulated wrist flexion angle peaks for all 25 participants.

**Figure 10:**
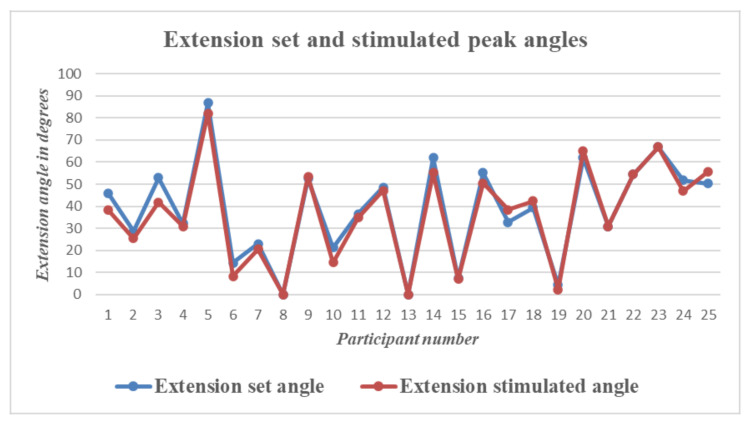
Wrist Extension—Graph showing differences between set and stimulated wrist extension angle peaks for all 25 participants.

The graph in Figure 9 shows a plot of the peak wrist flexion values for the set and stimulated values obtained for each participant. Similarly, Figure 10 shows a plot of the peak extension set and stimulated values obtained for each participant.

## 5. Discussion

The aim of this study was to enable bilateral symmetrical mirrored movement wrist flexion and extension by providing visual, sensory feedback and control wrist movement with functional electrical stimulation. It was observed that proper placement of electrodes and stimulation–angle calibration are essential to achieving good, symmetrical and in-phase movements.

In a few subjects, placement of the electrodes for symmetrical flexion and extension was very difficult. It was also observed that in some subjects, flexion of the wrist occurred while the electrodes were stimulating the extensors. This was probably due to the larger loop current pathway that was stimulating the wrist flexors. Here, moving the electrodes closer to each other for a more superficial stimulation current path rectified the problem. In two subjects, the electrode placements caused thumb extension alone and no wrist or finger extension. When the palm of the hand was used to grasp the handle of the encoder, with the thumb in opposition to the rest of the fingers and stimulated, the thumb was being extended, thereby pulling the other fingers into flexion and moving the encoder handle in the opposite direction. The electrodes had to be moved around, and the palm of the hand was placed on one side of the encoder handle and strapped into position. For some participants, the forearm and wrist belt described in [42] proved useful to locate the position of the electrodes a second time.

In a few participants, (three during flexion and four during extension), perfect angle coordination was achieved, but in the majority, there were slight variations between the set and stimulated angles.

In cases where the stimulated angle was lower than the set angle, it was seen that the set angle was set to go beyond tabulated stimulation values and, therefore, remained at the last stimulation point. In the cases where the stimulation peaks were higher than set values, as can be seen in the colored rows in the table, it was assumed to be either due to baseline errors during calibration or some other factors. During calibration, stimulation pulses last for 6 s, after which there is a break of 6 s. At the end of the stimulation time, during the break, since the wrist is not being stimulated and muscles are relaxed, the encoder swings back to the baseline and in some rare cases, below the baseline. When this happens, the value of the next angle recorded will be slightly lower than the actual angle value. Presumably, this could be the reason for the error. Two participants were nervous and resisted movement during stimulation. This was another reason for errors during set/stimulate and record.

**Figure 11:**
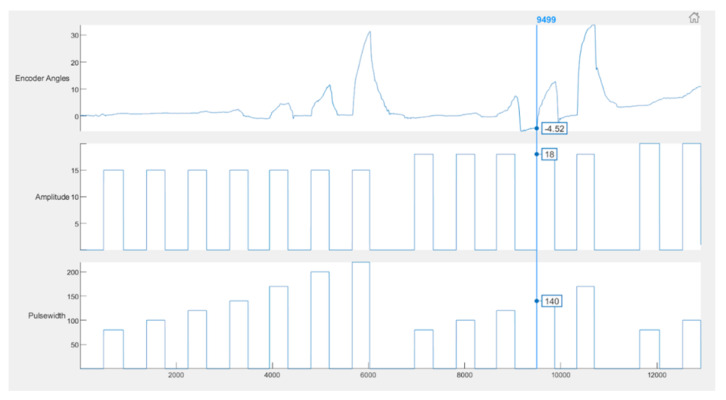
Example of error during calibration. The vertical line passing through the graph shows that at 18 mA and 140 µs, the baseline value was −4.52 instead of 0, which is one possible source of error.

In the Figure 11, an example of this error is shown. The baseline has been shifted below 0 to a value −4.52. The peak angle is measured and saved as 12.35, whereas the actual value is 12.35 + 4.52 = 16.87 degrees. In most cases, the shifted baseline is negligible and barely causes any issues.

The stimulated angle curve lags behind the set angle curve by a mean time of 1.02 s, which is negligible for therapeutic and clinical purposes. The cause of this lag is largely due to controller gain and minimally due to the latent period between start of stimulation and onset of muscle contraction.

A few participants found stimulation and record angles to be a little uncomfortable at times. For these participants, a third recording was performed with a fixed amplitude and changing pulse widths. The look-up table was checked and sorted based on the ascending order of amplitude. For each amplitude, the range of angle values was checked and the one that had the highest angle range and largest number of changing pulse widths was chosen. The stimulate and record angles app was run with this angle table. Participants on this recording found stimulation to be much more comfortable, and on examining the curves of both encoders, the lag between the set and stimulated angles was negligible.

The graphs show that the stimulate angle follows the set angle very closely, with negligible difference between the two sides and negligible time lag. In stroke, being able to move the wrist flexors and extensors on the paralyzed side with electrical stimulation controlled by the normal side has many advantages. Since the activity is bilateral, there is some amount of synchronization of motor intent with activation of the target muscle. Visual feedback of the limb moving and sensory/motor feedback due to stimulation are other factors that might have a huge influence on re-learning the activity by a different part of the brain.

This study was performed to evaluate the efficiency of the device and to determine how closely wrist flexion/extension was being mirrored onto the other side. Since the results of this study are quite promising, future studies will be performed on stroke patients to evaluate the amount of re-learning taking place. In this study, only a single degree of freedom (DOF), namely wrist flexion and extension, was considered. The addition of more DOFs will increase the versatility of the device and improve results.

## 6. Conclusions

It has been demonstrated that wrist extension and flexion can be mirrored without the use of a mirror by simply using angle-controlled delivery of electrical stimulation. The results show that when there is a wide enough range of stimulation parameters for moving the wrist between 60° extension and 60° flexion, the stimulated wrist can follow the set side wrist. FES was used to stimulate the wrist muscles and mirror wrist flexion/extension. To take this research further, trials will be conducted on stroke patients to evaluate the usability and feasibility of this device in a clinical setting.

## Figures and Tables

**Table 1:** Example of an extension table. The maximum encoder angle obtained for every increase in amplitude and/or pulse width of stimulation are recorded along with amplitude and pulse width values in a table called the calibration table.

S. No.	Angle	Amplitude	Pulse Width
1.	−0.7000	18	80
2.	−0.4300	18	100
3.	1.9540	15	120
4.	2.8925	20	80
5.	3.0520	18	120
6.	3.4545	15	80
7.	4.3500	15	140
8.	4.5335	24	80
9.	4.9555	20	100
10.	10.6625	15	170
11.	11.7900	15	100
12.	30.0085	18	140
13.	30.0725	15	200
14.	32.7865	24	100
15.	35.9210	28	80
16.	41.6605	20	120

**Table 2:** Mean errors with time lag.

Flexion (Angle in Degrees)			Extension (Angle in Degrees)			Time (in MS)		
		Error			Error			Lag
Mean		2.3668	Mean		1.908	Mean		1016.4
Std. deviation		5.38	Std. deviation		3.95	Std. deviation		492.09

## Data Availability

Data supporting reported results can be obtained on requesting the corresponding author.

## References

[1] The Lancet: Latest Global Disease Estimates Reveal Perfect Storm of Rising Chronic Diseases and Public Health Failures Fuelling COVID-19 Pandemic|Institute for Health Metrics and Evaluation. https://www.healthdata.org/news-release/lancet-latest-global-disease-estimates-reveal-perfect-storm-rising-chronic-diseases-and.

[2] Waller S.M., Whitall J. (2008). Bilateral arm training: Why and who benefits?. NeuroRehabilitation.

[3] Sethy D., Sahoo S., Kujur E.S., Bajpai P. (2018). Stroke upper extremity rehabilitation: Effect of bilateral arm training. Int. J. Health Allied Sci..

[4] Chen P.M., Kwong P.W.H., Lai C.K.Y., Ng S.S.M. (2019). Comparison of bilateral and unilateral upper limb training in people with stroke: A systematic review and meta-analysis. PLoS ONE.

[5] Lee M.J., Lee J.H., Koo H.M., Lee S.M. (2017). Effectiveness of Bilateral Arm Training for Improving Extremity Function and Activities of Daily Living Performance in Hemiplegic Patients. J. Stroke Cerebrovasc. Dis..

[6] Sainburg R.L., Good D., Przybyla A. (2013). Bilateral Synergy: A Framework for Post-Stroke Rehabilitation. J. Neurol. Transl. Neurosci..

[7] Swinnen S.P. (2002). Intermanual coordination: From behavioural principles to neural-network interactions. Nat. Rev. Neurosci..

[8] Cohen L. (1971). Synchronous bimanual movements performed by homologous and non-homologous muscles. Percept. Mot. Skills.

[9] Kelso S.J., Southard D.L., Goodman D. (1979). On the Nature of Human Interlimb Coordination. Science.

[10] Stoykov M.E., Corcos D.M. (2009). A review of bilateral training for upper extremity hemiparesis. Occup. Ther. Int..

[11] van Delden A.E.Q., Peper C.E., Kwakkel G., Beek P.J. (2012). A Systematic Review of Bilateral Upper Limb Training Devices for Poststroke Rehabilitation. Stroke Res. Treat..

[12] Brackenridge J., Bradnam L.V., Lennon S., Costi J.J., Hobbs D.A. (2016). A Review of Rehabilitation Devices to Promote Upper Limb Function...: Ingenta Connect. Neurosci. Biomed. Eng..

[13] Nudo R.J. (2013). Recovery after brain injury: Mechanisms and principles. Front. Hum. Neurosci..

[14] Kimberley T.J., Samargia S., Moore L.G., Shakya J.K., Lang C.E. (2010). Comparison of amounts and types of practice during rehabilitation for traumatic brain injury and stroke. J. Rehabil. Res. Dev..

[15] Sousa A.S.P., Moreira J., Silva C., Mesquita I., Macedo R., Silva A., Santos R. (2022). Usability of Functional Electrical Stimulation in Upper Limb Rehabilitation in Post-Stroke Patients: A Narrative Review. Sensors (Basel).

[16] Chae J., Bethoux F., Bohinc T., Dobos L., Davis T., Friedl A. (1998). Neuromuscular stimulation for upper extremity motor and functional recovery in acute hemiplegia. Stroke.

[17] Kimberley T.J., Lewis S.M., Auerbach E.J., Dorsey L.L., Lojovich J.M., Carey J.R. (2004). Electrical stimulation driving functional improvements and cortical changes in subjects with stroke. Exp. Brain Res..

[18] Powell J., Pandyan A.D., Granat M., Cameron M., Stott D.J. (1999). Electrical stimulation of wrist extensors in poststroke hemiplegia. Stroke.

[19] Sharififar S., Shuster J.J., Bishop M.D. (2018). Adding electrical stimulation during standard rehabilitation after stroke to improve motor function. A systematic review and meta-analysis. Ann. Phys. Rehabil. Med..

[20] Wu F.C., Lin Y.T., Kuo T.S., Luh J.J., Lai J.S. (2011). Clinical effects of combined bilateral arm training with functional electrical stimulation in patients with stroke. IEEE Int. Conf. Rehabil. Robot..

[21] Cauraugh J.H., Kim S.B., Duley A. (2005). Coupled bilateral movements and active neuromuscular stimulation: Intralimb transfer evidence during bimanual aiming. Neurosci. Lett..

[22] Knutson J.S., Hisel T.Z., Harley M.Y., Chae J. (2009). A Novel Functional Electrical Stimulation Treatment for Recovery of Hand Function in Hemiplegia: 12-Week Pilot Study. Neurorehabil. Neural Repair.

[23] Kang N., Idica J., Amitoj B., Cauraugh J.H. (2014). Motor recovery patterns in arm muscles: Coupled bilateral training and neuromuscular stimulation. J. Neuroeng. Rehabil..

[24] Xiao L., Yu Z., Mao M. (2018). Contralaterally controlled functional electrical stimulation improves wrist dorsiflexion and upper limb function in patients with early-phase stroke: A randomized controlled trial. Ann. Phys. Rehabil. Med..

[25] Chan M.K., Tong R.K., Chung K.Y. (2009). Bilateral Upper Limb Training With Functional Electric Stimulation in Patients with Chronic Stroke. Neurorehabilit. Neural Repair.

[26] Kitamura T., Sakaino S., Tsuji T. Bilateral control using functional electrical stimulation. Proceedings of the IECON 2015-41st Annual Conference of the IEEE Industrial Electronics Society.

[27] Gandhi D.B.C., Sterba A., Khatter H., Pandian J.D. (2020). Mirror Therapy in Stroke Rehabilitation: Current Perspectives. Ther. Clin. Risk Manag..

[28] Samuelkamaleshkumar S., Reethajanetsureka S., Pauljebaraj P., Benshamir B., Padankatti S.M., David J.A. (2014). Mirror Therapy Enhances Motor Performance in the Paretic Upper Limb After Stroke: A Pilot Randomized Controlled Trial. Arch. Phys. Med. Rehabil..

[29] Kim K., Lee S., Kim D., Lee K., Kim Y. (2016). Effects of mirror therapy combined with motor tasks on upper extremity function and activities daily living of stroke patients. J. Phys. Ther. Sci..

[30] Kim H., Lee G., Song C. (2014). Effect of Functional Electrical Stimulation with Mirror Therapy on Upper Extremity Motor Function in Poststroke Patients. J. Stroke Cerebrovasc. Dis..

[31] Lin K., Huang P., Chen Y., Wu C., Huang W. (2014). Combining Afferent Stimulation and Mirror Therapy for Rehabilitating Motor Function, Motor Control, Ambulation, and Daily Functions After Stroke. Neurorehabil. Neural Repair.

[32] Mathieson S., Parsons J., Kaplan M. (2014). Combining functional electrical stimulation with mirror therapy for the upper limb in people with stroke. Crit. Rev. Phys. Rehabil. Med..

[33] Gera S., Gangadharan N., Navin B.P., Tharion G., Chalagiri P.H., Thomas R., Devasahayam S. Electrical Stimulation and assessment of the induced force in the Denervated Muscle. TENCON 2019 - 2019 IEEE Region 10 Conference (TENCON).

[34] Nehrujee A., Andrew H., Patricia A., Samuelkamaleshkumar S., Prakash H., Sujatha S., Balasubramanian S. (2021). A Plug-and-Train Robot (PLUTO) for Hand Rehabilitation: Design and Preliminary Evaluation. IEEE Access.

[35] Mortimer J.T., Bhadra N. (2004). Chapter 4.2 Peripheral Nerve and Muscle Stimulation. Neuroprosthetics Theory and Practice.

[36] Gobbo M., Maffiuletti N.A., Orizio C., Minetto M.A. (2014). Muscle motor point identification is essential for optimizing neuromuscular electrical stimulation use. J. Neuroeng. Rehabil..

[37] Lyons G.M., Leane G.E., Clarke-Moloney M., O’Brien J.V., Grace P.A. (2004). An investigation of the effect of electrode size and electrode location on comfort during stimulation of the gastrocnemius muscle. Med. Eng. Phys..

[38] Flodin J., Juthberg R., Ackermann P.W. (2022). Effects of electrode size and placement on comfort and efficiency during low-intensity neuromuscular electrical stimulation of quadriceps, hamstrings and gluteal muscles. BMC Sports Sci. Med. Rehabil..

[39] Medeiros F.V.A., Vieira A., Carregaro R.L., Bottaro M., Maffiuletti N.A., Durigan J.L.Q. (2015). Skinfold thickness affects the isometric knee extension torque evoked byNeuromuscular Electrical Stimulation. Brazilian J. Phys. Ther..

[40] Keller T., Kuhn A. (2009). Skin properties and the influence on electrode design for transcutaneous (surface) electrical stimulation. IFMBE Proc..

[41] Petrofsky J. (2008). The effect of the subcutaneous fat on the transfer of current through skin and into muscle. Med. Eng. Phys..

[42] Solomons C.D., Shanmugasundaram V. Forearm and wrist band for Functional Electrical Stimulation. Proceedings of the 2019 Innovations in Power and Advanced Computing Technologies.

